# Bacterial ‘Grounded’ Prophages: Hotspots for Genetic Renovation and Innovation

**DOI:** 10.3389/fgene.2019.00065

**Published:** 2019-02-12

**Authors:** Bhaskar Chandra Mohan Ramisetty, Pavithra Anantharaman Sudhakari

**Affiliations:** Laboratory of Molecular Biology and Evolution, School of Chemical and Biotechnology, SASTRA Deemed University, Thanjavur, India

**Keywords:** genome plasticity, genome evolution, horizontal gene transfer, bacterial ecology, bacteriophage

## Abstract

Bacterial genomes are highly plastic allowing the generation of variants through mutations and acquisition of genetic information. The fittest variants are then selected by the econiche thereby allowing the bacterial adaptation and colonization of the habitat. Larger genomes, however, may impose metabolic burden and hence bacterial genomes are optimized by the loss of frivolous genetic information. The activity of temperate bacteriophages has acute consequences on the bacterial population as well as the bacterial genome through lytic and lysogenic cycles. Lysogeny is a selective advantage as the prophage provides immunity to the lysogen against secondary phage attack. Since the non-lysogens are eliminated by the lytic phages, lysogens multiply and colonize the habitat. Nevertheless, all lysogens have an imminent risk of lytic cycle activation and cell lysis. However, a mutation in the attachment sites or in the genes that encode the specific recombinase responsible for prophage excision could result in ‘grounding’ of the prophage. Since the lysogens with grounded prophage are immune to respective phage infection as well as dodge the induction of lytic cycle, we hypothesize that the selection of these mutant lysogens is favored relative to their normal lysogenic counterparts. These grounded prophages offer several advantages to the bacterial genome evolution through propensity for genetic variations including inversions, deletions, and insertions via horizontal gene transfer. We propose that the grounded prophages expedite bacterial genome evolution by acting as ‘genetic buffer zones’ thereby increasing the frequency as well as the diversity of variations on which natural selection favors the beneficial variants. The grounded prophages are also hotspots for horizontal gene transfer wherein several ecologically significant genes such as those involved in stress tolerance, antimicrobial resistance, and novel metabolic pathways, are integrated. Moreover, the high frequency of genetic changes within prophages also allows proportionate probability for the *de novo* genesis of genetic information. Through sequence analyses of well-characterized *E. coli* prophages we exemplify various roles of grounded prophages in *E. coli* ecology and evolution. Therefore, the temperate prophages are one of the most significant drivers of bacterial genome evolution and sites of biogenesis of genetic information.

## Introduction

**FIGURE 1:**
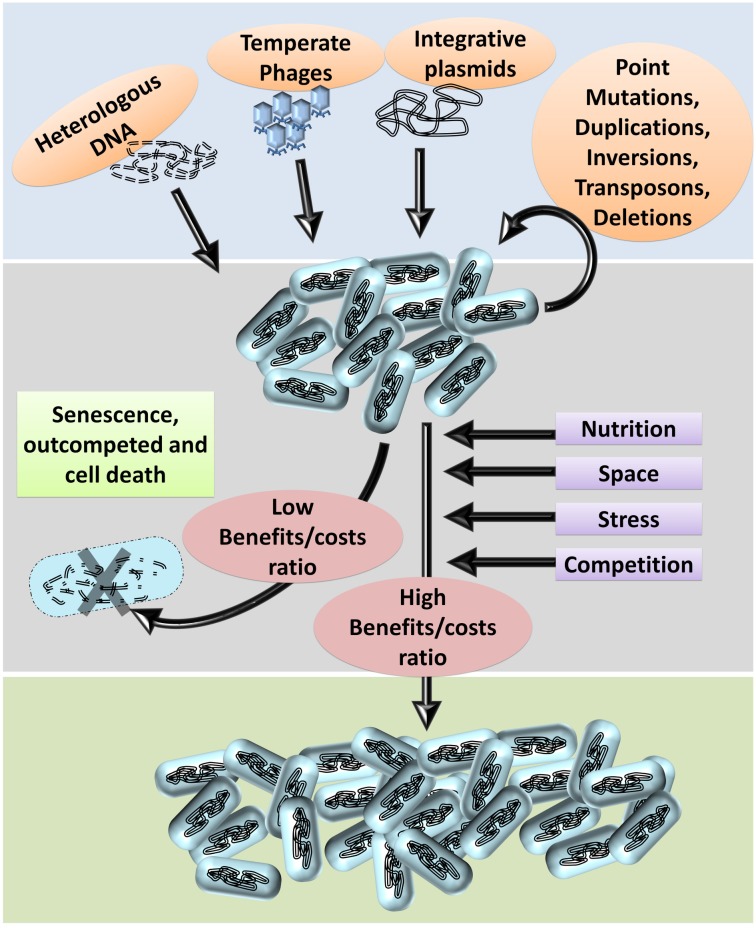
Bacterial genome evolution. Based on the habitat, bacterial genomes acquire DNA (genetic information) from the environment. Genetic information from the phages, plasmids, and dead organisms maybe integrated into the genome by homologous or non-homologous recombination.

Genome plasticity is an essential requirement for evolution directed by ‘econiche.’ Bacterial genomes are highly plastic relative to other organisms owing to the diversity and dynamicity of the niche (Dobrindt et al., 2010). The success of bacterial ‘omnipresence’ is predominantly dependent on the propensity and probability of genomic plasticity. The frequent exposure to and the ability to integrate exogenous genetic information enhances their genome plasticity (Hacker and Carniel, 2001; Dobrindt et al., 2010; Darmon and Leach, 2014; D’Souza et al., 2015). The basic principles of bacterial genomic plasticity comprise of (i) acquisition of exogenous genetic information and (ii) deletion of unnecessary genetic information at the population level by the selection imposed by the niche. The niche, including the inter and intraspecies competition, is the prime factor that imposes the selection of beneficial genetic information and optimization of the genomes based on the costs and benefits of each variation (Figure 1). The intraspecies competition achieves deletion of non-beneficial genetic information wherein the individuals with a higher metabolic burden, and slow growth rate are eliminated under natural testing conditions thereby selecting individuals with lesser ‘junk’ DNA (Dobrindt and Hacker, 2001). Furthermore, bacteria with deleterious recombination events or mutations are also eliminated thereby gradually ‘ridding off’ of frivolous genetic information from the population through a process generally referred to as ‘purifying selection.’ In other words, the econiche selects the best ‘composites of genetic information’ of the available ones based on the reproductive potential and stress tolerance imposed by the habitat (Forde et al., 2004).

Genome plasticity is predominantly mediated by horizontal gene transfer (HGT) and intragenomic recombinations such as transposition. The three principal HGT mechanisms are (i) transformation (direct uptake of DNA), (ii) transduction (DNA transfer mediated by phages), and (iii) conjugation (DNA transfer through physical contact between two bacteria) (Davison, 1999; von Wintersdorff et al., 2016). Of the three HGT mechanisms, transduction is highly ‘active’ by virtue of viral tropism, and mechanisms of DNA injection into the recipient bacterium. Occasionally, some of the temperate phages reversibly integrate into the host genome by a phenomenon referred to as lysogeny. The integrated phage genome, now referred to as ‘prophage,’ is vertically inherited by the daughter cells; the prophage replicates as a part of the host genome. Eventually, the prophage would switch from lysogenic to lytic cycle often resulting in phage multiplication and host cell death (Gandon, 2016). Bacteriophages, in general, play an important role in the ecology as well as the evolution of bacteria through several types of interactions between the host and phage genomes (Roossinck, 2011; Nasir et al., 2017). In fact, phages and their hosts have coevolved strategies that impact the survival, persistence, and evolution of their respective genomes (Buckling and Brockhurst, 2012; Koskella and Brockhurst, 2014; Nasir et al., 2017). Prophages were shown to influence the microbial community through enhanced recombinations (Nadeem and Wahl, 2017; Braga et al., 2018).

Mutations in one or more genetic elements required for the excision result in the failure of prophage excision from the host genome rendering them ‘grounded,’ also referred to as ‘cryptic’ or defective prophages (Casjens, 2003; Wang et al., 2010). The whole genome sequencing of diverse bacterial species has revealed multiple prophages within each genome. The prevalence of multiple grounded prophages within most bacterial genomes is indicative of eco-evolutionary selection of such genomes. Although the advantages of prophages are being explored (Bobay et al., 2014; Menouni et al., 2015; Gandon, 2016; Howard-Varona et al., 2017), the ecological scenarios and the possible evolutionary impact of these prophages are still unclear. In this manuscript, we discuss the mechanisms of grounding, and propose eco-evolutionary perspectives rationalizing the prevalence and advantages of grounded prophages to the host bacteria. We performed sequence analyses of five grounded prophages (DLP12, e14, Rac, CPZ-55, and Qin) of *Escherichia coli* to illustrate the distribution and the advantages.

Furthermore, several types of mutations can occur in the genome such as point mutations, duplications, inversions, deletions, etc. Within a population of bacteria, these genetic variations occur at a low frequency. Often, these variations are deleterious: i.e., the benefits caused by the variations are less than the costs incurred. Several parameters, such as nutrition, stress tolerance, competition, and resistance to antibiotics impose stringent selective pressure on the population. Hence, bacteria with deleterious variations usually perish or are outcompeted by the faster growing kin. Bacteria with beneficial genetic variations, such as higher growth rates, novel metabolic pathways, higher tolerance to stress, and higher competitive fitness fare better and are selected.

Each habitat may pose various stresses of varying degrees, allowing the gradual selection of organisms optimal for successful propagation in the prevailing conditions.

## Bacterial Genome Plasticity

Genome plasticity drives the successful spatiotemporal propagation of bacteria in terms of ecology as well as evolution. Genetic rearrangements, caused by transformation, transposition, and recombination events (site-specific as well as homologous), enhanced by insertion sequences (IS) elements, integrons, conjugative transposons, plasmids, bacteriophages, pathogenicity islands, and genomic islands result in a gain (or loss) of genes with diverse functions (Dobrindt and Hacker, 2001; Betts et al., 2018). Different bacteria may have different degrees of plasticity (Gaudriault et al., 2006) and, furthermore, regions such as ‘Regions of Genomic Plasticity’ (RGPs) of a bacterial genome are thought to have a higher propensity for genetic rearrangements relative to other core regions. The four different RGPs categories (genomic islands, prophages, regions encoding recombinases, and unclassified) are mosaics consisting of several modules. Each module ranging from 0.5 to 60 kb consists of genes involved in metabolism, intra and intercellular DNA mobility, drug resistance, host/environmental interactions, and antibiotic synthesis (Ogier et al., 2010). Different bacterial species have varying potential to take up exogenous DNA, metabolize it as a nutrient, or recombine the incoming DNA into the genome. The ability to take up exogenous DNA is termed as competence, a complex mechanism of temporal regulation for the uptake as well as the integration of the exogenous DNA (Blackstone and Green, 1999; Palchevskiy and Finkel, 2006; Maughan et al., 2010; Sinha and Redfield, 2012; Mell and Redfield, 2014). Often, the DNA could be catabolized and reused in nucleic acid metabolism or for generation of energy depending on the metabolic state of the cell (Finkel and Kolter, 2001). The DNA that is taken up maybe recombined into the bacterial host genome by homologous recombination or non-homologous mechanisms resulting in transformation (Fischer et al., 2001; Blokesch, 2017; Veening and Blokesch, 2017). Integration of exogenous DNA is the largest contributor of the pan-genome, the cumulative genetic information of all strains of a single bacterial species. The total amount of incoming DNA during the existence of a species in a habitat, theoretically, would imply an enormous genome. However, bacterial populations tend to get rid of larger genomes by intraspecies competition in limiting conditions imposed by the niche. The reduction of genome size is evident from the occurrence of multiple pseudogenes and loss of several genes within the genomes of most bacteria (Lawrence et al., 2001).

A typical bacterial species has expanded the horizons of its habitat through genome plasticity. When non-pathogenic bacteria infect a host, the niche may select the individual bacterium that may have specific mutations/genetic information allowing survival/virulence and eventually evolve into a pathogenic strain (Hogardt et al., 2007). Such mutations confer fitness by the acquisition of virulence genes and loss of antivirulence genes that nullify the action of factors conferring virulence (Dobrindt et al., 2010). For example, the presence of antivirulence genes encoding enzymes involved in arabinose catabolism has reduced the virulence in non-pathogenic *Burkholderia thailandensis*. This has been experimentally shown wherein *B. pseudomallei* that harbors arabinose assimilation operon, grown with arabinose as the only carbon source showed *Salmonella*-like gene cluster down-regulation (TTSS3-Type III secretion system) involved in virulence (Moore et al., 2004). *Shigella* and enteroinvasive *E. coli* (EIEC) species have acquired their virulence through structural mutation of the *cadA* gene. Another mechanism involved in pathoadaptation is the mutation of *cis*-regulatory elements (CRE). For example, mutational study in *Salmonella* species, on the role of *cis*-regulatory elements, revealed that evolutionary mutations in *cis*-regulatory elements such as SsrB regulatory system enhanced the expression of *srfN* gene involved in intrahost fitness (Osborne et al., 2009). The enormous genetic variability in *Helicobacter pylori*, a human pathogen, shows the extent of adaptation brought about by HGT and homologous recombination. However, these sequence variations have occurred without a drastic increase in the genome size of the bacterium (Kraft et al., 2006). Among closely related *Salmonella* serotypes, extensive genetic diversity was observed in the antigenic determinant genes that encode factors responsible for lipopolysaccharides (LPS), flagella, fimbriae and virulence factors (Fierer and Guiney, 2001). Genome plasticity allows oscillation of acquisition and loss of genetic information from the population as per the change in growth conditions and the habitats.

## Prevalence of Prophages in Bacterial Genomes

It is conceivable that the prophages are the largest exogenous contributors of bacterial genetic information, primarily, because of their sheer size. Astonishingly, prophages may constitute 10–20% of a bacterial genome predominantly due to an exposure to a multitude of bacteriophages (Casjens, 2003). Of the completely sequenced *E. coli* strains, *E. coli* O157:H7 str. Sakai has the highest number of prophages per genome; it harbors 18 prophage elements constituting 16% of the total genome (Asadulghani et al., 2009). Often, these prophages could be activated by several stresses to undergo lytic cycle. For example, 40 out of 148 *Lactobacilli* strains, representing 15 species, released phage particles upon mitomycin induction. In particular, 77% of 30 *L. Salivarius* produced temperate phages, killer particles or defective phages. Temperate prophages were found in 10% of 105 strains of *L. bulgaricus* and *L. lactis* (Sechaud et al., 1988). Using VirSorter tool (Roux et al., 2015a), around 5,492 microbial genomes out of the 14,973 publicly available bacterial and archaeal genomes (as on January 2015) had viral-like elements of which 2,445 contained more than one detectable phage elements. 82% of such phage co-infections involved multiple *Caudovirales* (Roux et al., 2015b). Bacterial genomes may contain intact prophages, LT2 isolate of *Salmonella enterica* (four prophages), pathogenic *Streptococcus pyogenes* isolate SF370 (prophage SF370.1) (Canchaya et al., 2002), *H. influenza* Rd (Flu-Mu) and *E. coli* sakai (Sp18) or defective prophages like two non-inducible prophages (Lp1 and Lp2) and two prophage remnants (R-Lp3 and R-Lp4) of *Lactobacillus plantarum* (Ventura et al., 2003). A large number of IS elements that are collectively referred to as minichromosomes comprise highly divergent prophage remnants constituting 22% of *Halobacterium* sp. NRC-l genome, and code for transposases associated with phage genomes and other mobile genetic elements (Bonneau et al., 2004). A recent web based tool called PhageWeb, for the identification and characterization of prophages within bacterial genomes, may expand the exploration of prophages in a variety of species (Sousa et al., 2018). The prevalence of prophages in bacterial genomes is intriguing, firstly, because of the imminent danger of lytic cycle induction and, secondly, the metabolic burden of replicating and expressing the prophage genetic elements. The eco-evolutionary rationales that operate to sustain prophage rich genomes of bacterial populations in the environment are poorly understood. It is indeed interesting to understand the beneficiaries of lysogeny: the host bacterial genome or the phage genome or both.

## Why Are Prophages Prevalent in Bacterial Genomes?

### (a) Lysogeny Mechanisms

**FIGURE 2:**
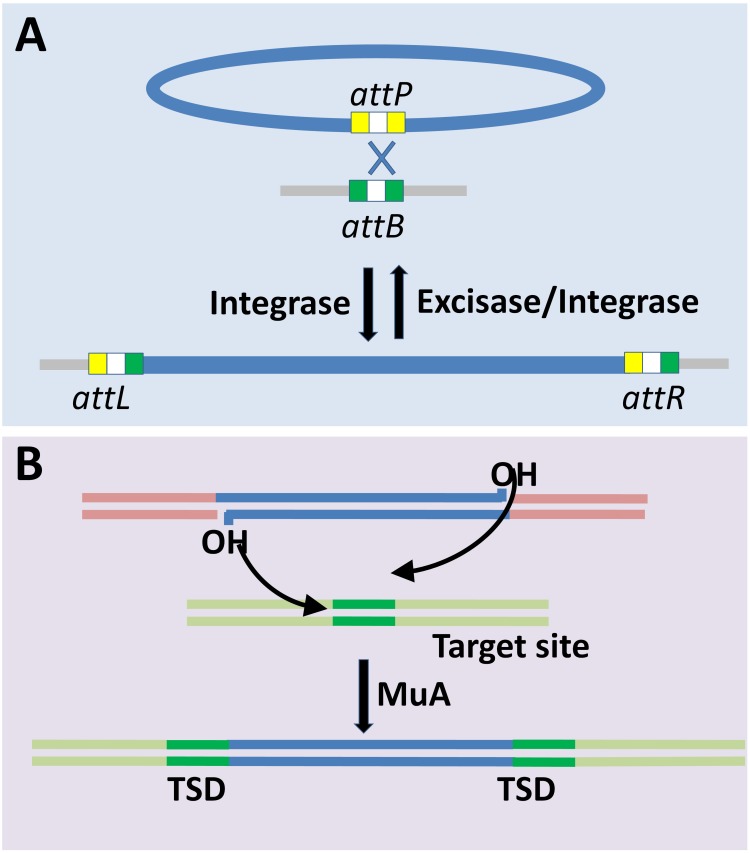
Mechanisms of bacteriophage genome integration into bacterial chromosome. **(A)** Site-specific recombination mediated-lysogenization is well characterized for lambdoid phages. Site-specific recombination is catalyzed by the integrase through phage and bacterial attachment sites (*attP* and *attB*, respectively) resulting in recombinant *attL* and *attR* sites (Landy and Ross, 1977; Menouni et al., 2015). Prophage induction is mediated by excisase and integrase complex. **(B)** Transposition based integration of Mu phage into the target site in the host genome requires MuA transposase. The integration results in target site duplication (TSD) (Allet, 1979; Harshey, 2014).

Lysogeny is a common feature in almost all habitats including the gut microbiome (Howard-Varona et al., 2017; Kim and Bae, 2018). Integration into the host genome is the fundamental step for the establishment of lysogeny. Different phages employ different mechanisms (Figure 2) that broadly fall into (i) site-specific recombination (Landy and Ross, 1977), and (ii) transposition based integration (Allet, 1979; Harshey, 2014). Site-specific recombination is a well-characterized mechanism of phage (specifically lambda bacteriophages) genome integration and is the only one discussed here for brevity. Site-specific recombination requires phage *attP* and host *attB* sites that share 15 nucleotides in common, and thus the crossover occurs either within or flanking regions of this core region (Landy and Ross, 1977). Despite defective *attB* site Int-mediated recombination occurs between *attP* and secondary attachment sites in the host genome, albeit at a lower frequency (Shimada et al., 1975). The Cre- and Int-dependent recombination between the *lox* sites are mediated by site-specific recombination system of P1 and λ phages, respectively. The *loxP* has two 17 bp binding site for Cre, and each of the sites has 13 bp inverted repeat and a 4 bp region which together forms 8 bp spacer region flanked by two 13 bp inverted repeats. The *loxP* and *loxB* sites share limited homology, and hence their recombination efficiency is altered with sequence variation. Cre recombinase makes 6 bp long double strand staggered cut in the spacer region, localized between the adjacent *loxP* sites followed by strand exchange. The process of Cre-mediated cleavage involves phosphodiester bond breakage at 3’ end and covalent interaction of the primed phosphate with the protein (Hoess and Abremski, 1985). A similar rejoining mechanism is mediated by λ Int recombinase (Craig and Nash, 1983). Four recognition sites form a synapse prior to the cleavage, and this process is homology dependent (Ross et al., 1979). In essence, the essentials required for integration or excision are the *att* sites (on the bacterial as well as the phage genomes) and the specific recombinase that recognizes the concomitant recombinase recognition sites present within the corresponding *att* sites. Different recombinases, or here integrases, recognize different sequence specificities wherein the recognized sequences may have considerable sequence differences. Cross-activity of a recombinase of one phage with *att* site of another is proportional to the degree of homology between their integrases and similarity between the core- and arm-type sites in attachment sites of the respective phages (Groth and Calos, 2004).

### (b) Hotspots for Prophage Integration

The fundamental premise is that any genome with deleterious recombination events, concerning the niche conditions, will be lost in the population whereas tolerable recombination events are unaffected. Occasionally, advantageous recombination events would be selected and that would eventually outcompete their kin. Integration of most prophages is by site-specific recombination at *att* sites or by transposition at a random site. Although there is a significant chance for the occurrence of the *att* sites at multiple loci of a bacterial genome, many integration events could be deleterious for the host genome because of gene disruptions or dysregulations leading to greater fitness costs. Various analyses of lysogens have shown that prophages are predominantly found at loci closer to non-coding genes (e.g., tRNA genes), a few functional genes, and intergenic regions. Hence, these integration events should be assumed as either tolerable or advantageous in the growth conditions of the context. For example, in *Ralstonia solanacearum* the *attB* site for ϕRSA1 prophage integration occurred downstream of the tRNA-Arg gene (Fujiwara et al., 2008) and in *E. coli*, the relatives of λ phage and P4 phage insert within the tRNA genes (Campbell, 2003). It is rational for a phage with a broader range of host specificity to prefer integration sites that are within protein-coding functional genes as their occurrence is conserved across distantly related species, subject to the tolerable consequences of the recombination (Hill et al., 1989). Based on the composition and length of the integration site, a variable number of sites within a host genome for the phage integration are possible. However, Mu-like prophages show random integration events due to transposition-based integration. A robust analysis of 471 *E. coli* and *Salmonella* prophages showed 58 distinctive integration loci for 369 *E. coli* prophages and 102 loci for *S. enterica* prophages (Bobay et al., 2013). Based on the empirical evidence of various prophages in numerous bacterial genomes, one may assume that integration of phage genome into that of the bacterial genome at the specified loci are either tolerable or advantageous in the ecological context in which these lysogens would be selected.

### (c) Advantages of Lysogeny

**FIGURE 3:**
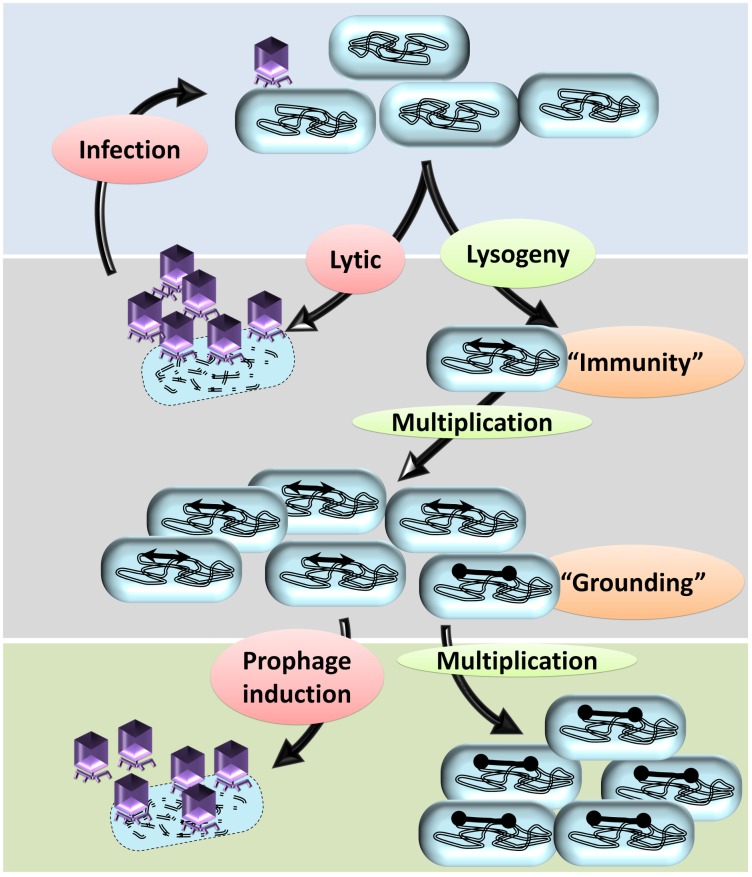
A hypothetical model to explain the prevalence of cryptic prophages in bacterial genomes. A typical temperate phage injects its genome in the bacterial cells that may undergo either lytic cycle or lysogeny. In the lytic cycle, more copies of phages are produced, the host bacterium is lysed, and a multitude of new phages are released into the surrounding. The lytic cycle repeats with each bacterium, which almost annihilates the bacterial population. In some bacteria, the injected genome undergoes lysogeny by integrating into the bacterial genome. Lysogeny allows vertical propagation of prophage along with that of the bacterial genome. By means of the prophage, the lysogens attain immunity to the infecting secondary phages thereby preventing infection/lysis. Apart from other mutants, the lysogens are the only surviving kin and hence will multiply well due to lack of intraspecies competition. Lysogeny, upon reversal, will ultimately lead to a lytic cycle leading to the death of the host bacterium. In some of the prophages, mutations in the recombinase recognition sites or recombinase genes occur, which prevent the elicitation of the lytic cycle: a phenomenon referred to as ‘grounding of prophages.’ The bacteria with grounded prophages have two advantages; immunity from attack by related secondary phages and irreversible lysogeny that prevents phage-mediated cell death. Eventually, other lysogens undergo lytic cycle upon induction by various stimuli resulting in lysis of the host bacterium. The bacterium with cryptic prophages will survive and have a growth advantage due to reduced competitive pressure from kin.

The size of the prophages (approximately 45 kb each) and the expression of prophage genes impose a significant metabolic burden on the lysogen growing in stringent natural conditions competing with non-lysogenic peers. It is conceivable that the bacterial cells, which do not harbor any prophages, have a competitive growth advantage relative to lysogens. However, as noted earlier, the prevalence of multiple cryptic prophages indicate that the lysogenic strains had a survival advantage compared to the non-lysogens. Intriguingly, lysogeny, as a trait of the bacterial genome as well as phage genome, must have been selected by virtue of some spatiotemporal advantage to both the host bacterium and the phage (Paterson et al., 2010; Roossinck, 2011). One of the straight forward explanations is that some of the prophages contribute genes of selective advantages, such as antibiotic resistance conferring genes encoding multidrug resistance pump, outer membrane protease, cell division inhibiting factors, small toxic membrane polypeptide, (Wang and Wood, 2016) etc., in the habitat and hence the lysogens would outcompete the non-lysogens. However, harboring large prophages that do not confer a selective advantage is a metabolic burden and hence is a fitness disadvantage to the lysogen. The key to deciphering this paradox—selection despite the metabolic burden—is the vulnerability of non-lysogens to phage-induced lysis while the lysogens are immune to the corresponding phage. Prophages bestow lysogens with immunity against secondary phage infections, referred to as superinfection exclusion (or immunity) (Figure 3). Assuming that the ecological niche of the bacterial population contains the phages, the lysogens have a survival advantage due to the ‘superinfection immunity’ conferred by the corresponding phage. Conferring immunity is ecologically vital for the resident prophage as well as the host bacterium. Lysogeny in conjunction with superinfection immunity confers the selective advantage to the lysogen from phage attack and concomitantly also allows the propagation of the lysogen. Superinfection immunity, as a mechanism to prevent multiple infections of a lysogen by similar phages, is achieved by different mechanisms such as: (i) conformational change in phage receptor sites, (ii) modifications in DNA transfer system, (iii) inhibition of phage lysozyme for penetration, (iv) incoming phage DNA breakdown, and (v) abortive exclusion. The gp15 expression of HK97 had shown resistance to HK97 and its close relative HK75 infections (Cumby et al., 2012). Similar factors include (i) SieA and SieB of *Salmonella* phage P22 blocks the DNA entry with unknown component of DNA transfer system and leads to abortive infection, respectively (Susskind et al., 1974), and (ii) Sie_2009_ of *Lactococcus lactis* phage Tuc_2009_ (Mahony et al., 2008). Rex system of λ prophage is thought to induce superinfection exclusion via abortive exclusion. RexA (a component of cell cytoplasm) is activated by protein–DNA interaction which then mobilizes RexB, a membrane ion channel protein, thereby altering the membrane potential of the host (Parma et al., 1992). The CI repressor (a positive regulator of its own expression) of the incumbent λ phage inhibits the establishment of superinfecting λ phage upon forming CI repressor-DNA complex and thus preventing the integration and replication of the secondary λ phage (Gottesman and Weisberg, 2004; Fogg et al., 2010). The defective e14 prophage-encoded Lit protease upon its activation by GoI peptide (located within the major head protein) cleaves the elongation factor EF-Tu, thus blocking the late gene expression of T4 phage (McGrath et al., 2002). Upon phages attack on a bacterial population, the only survivors are the lysogens and the spontaneously evolved mutants that are no longer recognized by the phage. Such scenarios would allow the elimination of the non-lysogens resulting in survival and growth of the lysogens due to extensive relaxation of peer competition for resources. Hence, the lysogens and the mutants will have unhindered access to the resources and the ‘spoils’ of the dead kin allowing the repopulation of the habitat, a phenomenon similar to that of bottlenecking.

It is also proposed that the prophage genes could confer a competitive advantage at a population level such as in the case of biofilm formation (Wang et al., 2010). The individual cost of prophage induction is explained as a mechanism of altruism since the induced lysis of the lysogens that benefits their population as a whole. For example, phage-induced lysis of the lysogens in the biofilm further enhances the biofilm formation by providing additional nutrients, bacterial dispersal, and extracellular DNA. Similarly, the bacteria competing for resources produce bacteriocins and require transport protein for their release. In cases where the transport protein is not encoded, like the group B colicins producing operon in *E. coli* and *Shigella*, the phage-induced lysis promotes the release of colicin bacteriocin (Obeng et al., 2016). Shiga toxin-producing *E. coli* are lysogens for lambdoid prophages that also encode Shiga toxin. The prophage induction is followed by the expression of Shiga toxin genes release of the Shiga toxin (Iversen et al., 2015). Shiga toxin targets the eukaryotic protein synthesis of cells that can internalize the toxin. In a small subset of the lysogens the prophage is induced to lytic cycle by stimuli like H_2_O_2_ (Shimizu et al., 2009; Los et al., 2010, 2013). The lysis of the bacteria causes the release of Shiga toxin which acts on their predators (protozoan or neutrophils as the case maybe) (Dydecka et al., 2017; Chakraborty et al., 2018). The attenuation or inactivation of the predators is thought to increase the survivability of the rest of the population. It is hypothesized that these Shiga converting prophages is an example of altruism at the population level. However, the concept of altruism is still debatable in the case of bacteria (Ramisetty et al., 2015; Durand et al., 2016; Peeters and de Jonge, 2018). Specifically with free-living bacteria, altruistic behavior would benefit the self and/or kin if only the beneficiaries of the donation are the kin. The ultimate altruism in the form of suicide is detrimental to the unicellular free-living species unless the fraction of donors is very small. Bacteria in nature occur as multispecies colonies/biofilms wherein intraspecies-altruism is highly unlikely because the sacrifice by the donors may benefit the competing species equally. Feeding the competitors is counterproductive to the goals of altruism. In the case of prophage-mediated host lysis, the selective induction of prophage, the mechanisms limiting the induction to a small fraction of the population, and the beneficiaries of the altruistic behavior require more experimental evaluation (Los et al., 2013).

Multiple other ecological advantages of lysogeny were also reported. Phage conversion upon transduction alters the host physiology with respect to metabolism, pathogenicity, and niche adaptation (Nasir et al., 2017). The integrated auxiliary metabolic genes (AMGs) among the marine viromes have been reported to carry essential genes for different nutrient cycles such as *dsr* and *sox* genesin sulfur cycle, *amok* and P-II in nitrogen cycle, photosynthesis (*psbA* and *psbD* genes from *Prochlorococcus* and *Synechococcus* cyanophages) (Roux et al., 2016), motility proteins (e.g., *flaB*), and flagellar motor complexes (e.g., *motA*) (Hurwitz et al., 2015). Prophages increase the survival (Steinberg and Levin, 2007), cell growth (Picozzi et al., 2015), virulence (Waldor and Mekalanos, 1996; Zschöck et al., 2000), promote biofilm formation, phase variation (Kutsukake and Iino, 1980; Silverman and Simon, 1980; Sekulovic and Fortier, 2015), quorum sensing (Hargreaves et al., 2014), confer resistance to antibiotics/environmental stresses (Hyder and Streitfeld, 1978; Lopez et al., 2012; Wipf et al., 2014), and immunotolerance (Farrant et al., 1997). Yet another example for prophage-enhanced environmental stress resistance of the lysogen is phage-regulated acid resistance in *E. coli*. Comparative analysis of non-lysogen and lysogen of Shiga-toxigenic phage (Φ24_B_), revealed the upregulation of operons encoding type I fimbriae and the glutamic acid decarboxylase (GAD) acid stress island (Veses-Garcia et al., 2015). It is highly likely that different prophages may have conferred one or more of the above discussed eco-evolutionary advantages. More such rationales could also be expected with the exploration and molecular examination of prophages in many other bacterial genomes. Of all the advantages discussed, immunity to superinfection is the most likely and effective advantage conferred by the prophage.

### (d) ‘Grounding’ of Prophages

Similar to transposons, the prophages are also prone to ‘grounding,’ which is to imply irreversible lysogeny due to genetic events that prevent their excision. Multiple *trans* and *cis* elements are involved in the site-specific recombination of the phage with the host genome. The site-specific integration of phage (specifically lambdoid bacteriophage) into their specific host genome via host and phage attachment sites (“*attB*” and “*attP*” sites, respectively) yields the recombinant “*attL*” and “*attR*” sites. *attL* and *attR* sites are the essential *cis* elements for excision of the integrated prophage upon switching from lysogenic to lytic cycle. The prerequisites for the integration and excision of phage genomes are (i) phage-encoded recombinase proteins (integrase and excisase); (ii) conserved prophage flanking “*attR*” and “*attL*” excisase recognition sites; and (iii) the expression of accessory host factors such as IHF and factor for inversion stimulation (Fis) proteins. Efficient excision is achieved by the cooperative role of both integrase and excisase. It has been shown that Xis protein promotes excision by recognizing the phage ‘*attR’* site in addition to sequence-specific binding of Int or IHF protein (Yin et al., 1985). The assembly of the excisive complex and the rate of prophage curing depends on the amount of Xis expressed in the host. The host Fis enhances the binding specificity of Xis thereby promoting the formation of excisive intasome on *attR* site (Papagiannis et al., 2007). The phage cII protein, along with IHF, upregulates the expression of Int and late gene repressors and thus promotes lysogeny. The mRNA level of the integrase gene in *S. aureus* prophage is influenced by the host alternative sigma factor σ^H^ (Tao et al., 2010). The Bxb1 prophage of *Mycobacterium* species encodes an accessory protein, gp47, which is involved in the directionality of Bxb1 integrase for the assembly of excisive intasome (Ghosh et al., 2006). Similarly, gp3 of *streptomyces* phage ΦBT (Zhang et al., 2013), gp52 of *streptococcus* bacteriophage ΦJoe, Rv1584c from ΦRv1 phage of *Mycobacterium tuberculosis* (Bibb et al., 2005), and SprA from SPβ prophage of *Bacillus subtilis* (Abe et al., 2014) are recombination directionality factors (RDFs). The insertion of the IS element and further rearrangement in lambdoid prophage of *Shigella dysenteriae* rendered them defective upon deletion of *stx* region (McDonough and Butterton, 1999). Similarly, the RNA-mediated translational control of *int* expression by *sib* results in the formation of a truncated integrase which in turn hampers the excision of defective prophages (Schindler and Echols, 1981).

The intracellular concentration of proteins involved in recombination such as Int, IHF, Xis, and Fis is highly regulated. IHF inhibition of excisive recombination occurs at lower levels of Int protein (Thompson et al., 1986). Hence, the mostly likely events for the grounding of temperate prophages are: (i) defective recombinase proteins (Swalla et al., 2003), (ii) homologous recombination during the super infection of related phages (Canchaya et al., 2003), and (iii) resected *att* sites. DNA invertase, Xis, and Fis could promote genetic rearrangement like DNA inversion, specifically between the *attP* site and secondary attachment site within the prophage. Such genetic rearrangements could also affect the expression of phage-encoded proteins and modify the orientation of the attachment sites resulting in the establishment of irreversible lysogeny or ‘grounding’ (Dorgai et al., 1993).

### (e) Degeneration of Prophages

The grounded prophages are highly prone to deletions, especially the genes whose products do not confer any advantage or are regressive. Harboring large prophages and expressing the prophage genes is a metabolic burden. Hence, minimizing such genetic elements are beneficial to the host genome. The closely related *E. coli* K-12 and *Shigella flexneri* showed variability in their prophage remnants (Brussow et al., 2004). A study on the genome diversity of *Salmonella enterica* serovar Typhimurium found deletion in *gipA* gene of L927 and L847 phages. Three complete deletions and four partial deletions in the virulence genes have been found in six of the sequenced strains of *Salmonella enterica*, emphasizing the diverse ecology of phage types. Study on *E. coli* O157:H7 EDL933 strain uncovered various types of indels of IS elements in 12 of their prophages (Iguchi et al., 2006). The prophages of *E. coli* O157 EDL933 have lost their deleterious gene such as λ N gene whose expression would result in host lysis. However, the neutral or beneficial genes for lysogenization like *int* and other phage structural genes occurred in most of their prophages. It was also found that *E. coli* K-12 harbored an incomplete Rac prophage, which was intact in EDL933 strain (Lawrence et al., 2001). A simulation study showed that large-scale deletions occurred in the accessory genes such as integrase and cargo genes, rather than the conserved genes involved in repression of lytic genes (Canchaya et al., 2002), replication, expression of capsid proteins, and packaging. For example, tail protein encoding regions are highly conserved over cargo genes in lambdoid prophages; morons 1 and 2 were least conserved in P2 like prophages (Bobay et al., 2014). The low ion-irradiation experiment showed that the IS elements and pseudogenes as a preferential site for deletions (Song and Luo, 2012) while a study on *Neisseria meningitides* strains showed a IS30 transposase replacement for head and tail morphogenesis genes (Dunning Hotopp et al., 2006). Deletion of inutile parts of the genome confers a growth advantage due to lowered metabolic burden or reduction in the inhibition of growth. Such bacteria will eventually outnumber the counterparts leading to smaller but optimal genomes in the population. However, other views suggest that the degeneration of prophages is driven by the need to eliminate the deleterious genes rather than to preserve chromosome compaction (Lawrence et al., 2001).

## What Are the Advantages of Grounded Prophages?

As noted earlier, prophages are dynamic zones within the genome wherein the frequency of genetic variation is very high. There are several disadvantages of bearing the grounded prophages, mostly stemming from the metabolic burden of replicating the large prophage and the expression of prophage genes. However, these disadvantages are subject to contextual economics, i.e., the resources available, growth conditions, and the competition would determine the costs to benefits ratio. The pre-selected lysogens continue to survive unless there are drastic changes in the growth conditions, competition or habitat change. There is also a possibility for the reversal of grounding through homologous recombination of a defective incumbent prophage with a related superinfecting-phage genome, leading to prophage activation and resulting in host lysis. Within a habitat, individual bacteria that may have acquired genetic changes within prophage imposing high costs/benefits are eliminated in the purifying selection. However, this purifying selection based on the prophage would have negligible impact on the species. On the other hand, there is a need to explore various rationales to reason the selection of individuals within a given habitat. There is minimal consistency in the preservation of a prophage. Besides, different prophages are implicated in a plethora of functions to the host genome. Hence, in the following sections we propose various advantages of harboring prophages to explain how prophages may have influenced the bacterial ecological success.

### (a) Variations due to Prophage Integration

Every event of integration causes a genetic variation; those that are either tolerable or advantageous are retained, similar to the consequences of transposition. If an integration event were deleterious, the bacterium with such mutations would be eliminated from the population. On the contrary, if a mutation is retained within the population, it must be either tolerable or advantageous. The λ integration at the *exuR* gene, involved in the regulation of *exu* regulon, rendered them non-functional in *E. coli* HfrH 58 strain. It is possible that the expression of *exu* regulon in the ecological context was not required. However, the functionality of *exuR* gene is regained upon prophage induction by site-specific recombination (Mata et al., 1978). Insertional inactivation of lipase-encoding gene by L54a and L54b prophages in *Staphylococcus aureus* PS54 has been reported. However, the curing of L54a prophage has restored the lipase-encoding gene expression but not with L54b curing (Lee and Iandolo, 1985). Similarly, *S. aureus* MW2 harbors ΦSa3mw prophage whose integration happened in haemolytic toxin gene, β-hemolysin (*Hlb*). Upon prophage induction, the *Hlb* gene expression was restored (Katayama et al., 2013). Most of the inversions occur as a result of homologous recombination between prophages (as hot spots for large-scale inversions), IS elements, and RNA-encoding operons (Iguchi et al., 2006; Camilli et al., 2011). Multiple inversions among the Mu-like prophages in various strains are shown to influence the host specificity by altering the expression of various host-cell-receptor recognition genes (Schmucker et al., 1986) and loss of tail genes (Braid et al., 2004). It is indeed difficult to ascertain if any of the phage integrations, solely by virtue of the integration event, has conferred a selective advantage.

### (b) Hotspots of Horizontal Gene Transfer

The probability of acquiring exogenous DNA into the genome is dependent on several aspects which include the availability of exogenous DNA, the competence of the host bacterium to take up the DNA, the expression of appropriate recombinases to integrate the DNA, and importantly the costs/benefits of the integration event. There is a lot of evidence that the numerous recombination events in a bacterial genome are associated with their prophages. Multiple prophages encode recombinases, which may aid in integration. However, the prevalence of indels within prophages is not indicative that prophages somehow ‘actively’ acquire genes. Rather, to state accurately, indels may occur randomly in any locus of the bacterial genome, but those recombinant genomes that have resulted in deleterious consequences (high costs) would have been eliminated from the population. Hence, the prevalence of multiple recombination events within the prophages of various strains within a given species implies that these recombinations are either tolerated or advantageous. This aspect could be best explained with the genetic phage mosaicism observed in several prophages. Phages of different groups or broad host range show the shuffling of virulence genes between their genomes (Desiere et al., 2001; Mirold et al., 2001). The number of mosaic phages is higher among gram negative bacteria over gram positive bacteria as the former have a broad host range and are recombinogenic (Brussow and Desiere, 2001).

Lambdoid prophages are capable of supplementing virion proteins to other lambdoid phages (Asadulghani et al., 2009). A study on the genome sequence of HK97, HK022, λ, and P22 prophages has found the mosaic pattern in their functional units such as individual genes, gene clusters, or a portion of protein-encoding genes (Juhala et al., 2000). Recombinases play a vital role in the formation of mosaics among prophages. For example, Rad52-like recombinase promoted the homologous recombination between lambdoid phages and *E. coli* prophage remnants. The prophage remnants in many cases have been found to complement functional genes involved in the lytic cycle. For example, DLP12 lysis protein essential in *E. coli* for the biofilm formation is also found active in lambda phages (De Paepe et al., 2014).

Horizontal gene transfer has led to the integration of ‘morons,’ homologs of bacterial genes, into the phage genomes. The moron gp15 of HK97 prophage in *Enterobacteriaceae* shared no homologs among the other phages but it did with bacterial genes that expressed YebO family of proteins. Other examples include GogB effector protein of *Salmonella enteric* prophage Gifsy-1 (Coombes et al., 2005) and NelA-like type III effector protein of enterohemorrhagic *E. coli* O157:H7 prophage BP4795 (Gruenheid et al., 2004). Similarly, *oac* moron of *Pseudomonas* phage D3 was homologous to *Pseudomonas* WbpC proteins involved in LPS biosynthesis (Burrows et al., 1996).

Prophages may confer direct selective advantages to their host. For example, the lysogenization of *E. coli* by CPS-53 and CP4-57 prophages promote host fitness under oxidative, osmotic, and acidic stresses (Wang et al., 2010). Likewise, RnlA toxin encoding gene in CP4-57 prophage (Koga et al., 2011) and CbtA toxin in CP4-44 (Tan et al., 2011) enhance persister phenotype in *E. coli* by inhibiting the cell growth. The paralogs of *nanS* esterase gene, required for the growth of pathogenic bacteria upon exploiting the host sialic acids, are found downstream of *stx* toxin genes in prophage or prophage remnants of *E. coli* strain O157:H7 (Rangel et al., 2016).

### (c) Hotspots of *de novo* Genesis

Since the generation of the genetic information and the evolution of the first cell, most of the genetic information must have evolved within cellular entities. Although it is possible for the *de novo* genesis of genes in bacterial cells, there is minimal tolerance for genetic events within the core gene-rich loci due to high costs and low benefits. The probability and tolerance for recombinations within the prophages serve as a platform for the generation of new genes. Hence, there is a relatively high probability for the *de novo* formation of new open reading frames (Bartels et al., 2009) and possibly selected if the product of the ORF is beneficial for the cell. With time and generations, the *de novo* formed gene may accumulate beneficial mutations and eventually be optimized for an appropriate function and regulated expression. For example, 94.2% of the genome of *Staphylococcus aureus* strain Jevons B contains ϕJB prophage that has 70 predicted ORFs (Bartels et al., 2009) out of which only 21 ORFs were associated with a putative function. The remaining 49 putative proteins have no known function, but structural domains and homologs have been identified for 26 and transmembrane region in 5 ORFs. The remaining proteins were recorded as “hypothetical” which refers to a legitimate ORF but encodes a protein with unknown function or association with any known proteins (Varga et al., 2016). A study on lysogeny module of StB27 prophage among *Staphylococcus hominis* and *Staphylococcus capitis* species found that 42% of the conserved proteins have no predicted functions, and 13 proteins shared no homologs. StB27 of coagulase-negative *Staphylococci* (CoNS) contained ORF3 of unknown function (Deghorain et al., 2012). Several ORFs of unknown function and hypothetical proteins have been found in prophages among *E. coli* O157 strains (Svab et al., 2013) (Saile et al., 2016). Similarly, numerous ORFs of unknown functions were identified in multiple species such as *Helicobacter pylori* (Luo et al., 2012), *Ralstonia solanacearum* strains (Askora et al., 2009), and *Pseudomonas aeruginosa* (Braid et al., 2004; Wang et al., 2004). P2-like coliphages of *Enterobacter* have ORFs that have hypothetical protein homologs in plasmids, genomes of other bacterial species (Nilsson et al., 2004). Interestingly, the prophage mEp021 of lysogenic *E. coli* K-12 harbors ORFs whose expression shows haemolytic activity in the host. The ORF-4 has four initiation codons within the single frame encoding for 83aa (ORF-4.1), 82aa (ORF-4.2), 77aa (ORF-4.3), and 72aa (ORF-4.4). Among these, the expression of ORF-4.3 has an inductive role in haemolytic activity by releasing vesicles containing bacterial protein HlyE and alters the morphology of the host bacterium (Martinez-Penafiel et al., 2012). The above examples reiterate that the great extent of the *de novo* genesis of novel genes is relative to any other sites on a typical bacterial genome. However, those ORFs whose products are functional and beneficial will be selected based on the costs/benefits and may evolve into beneficial genes with a high degree of propagative potential.

## Grounded Prophages of *E. coli*: Illustrative Analyses

**FIGURE 4:**
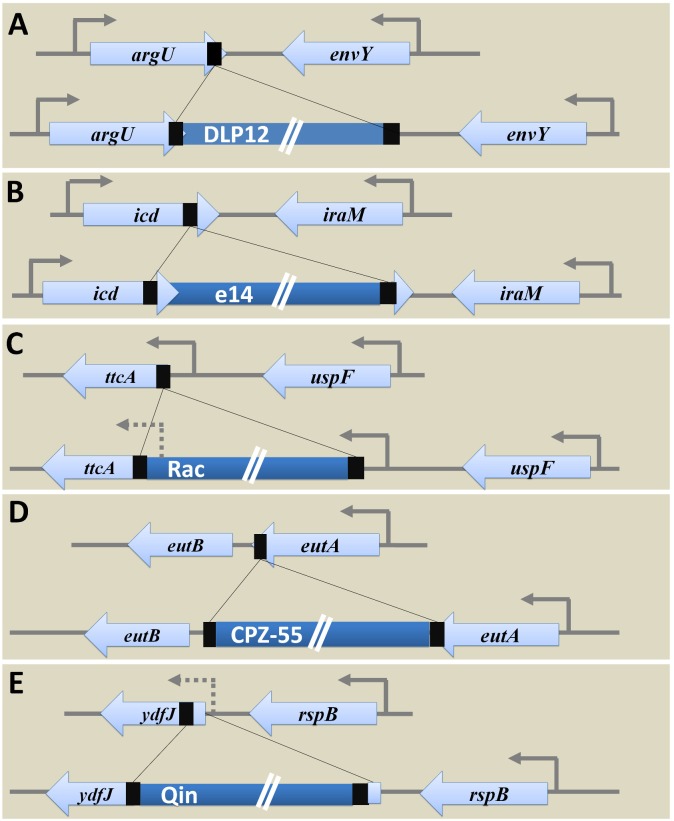
Genetic variations caused by prophage integration in *E. coli* MG1655. The examination of specific integration loci of each of the five prophages (DLP12, e14, Rac, CPZ-55, and Qin) concerning *E. coli* MG1655 among the strains with and without corresponding prophages uncovered the genetic variations caused post integration event as shown above. **(A)** Integration of DLP12 via homologous recombination near the 3′ end of the *argU* gene. The 47 bp region similar to 3′ end of *argU* gene is located on the other end of the DLP12. **(B)** Integration of e14 at the 3′ end of *icd* gene causing the formation of pseudo *icd’* (163 bp) gene as a duplicated region. **(C)** Site-specific integration of Rac at the 5′ end of *ttcA* gene. The variation in the N-terminus of TtcA protein was observed. **(D)** The transposon-like integration of CPZ-55 next to the stop codon of the *eutA* gene causing duplication of 8 bp region. No variations were seen in the bacterial flanking genes (*eutB* and *eutA*). **(E)** The site-specific integration of Qin at the 5′ end of *ydfJ* gene resulting in promoter loss and truncation (lacking 28 codons at the N terminal) of YdfJ protein.

*E. coli* K12 MG1655 strain has nine cryptic prophages of which DLP12 (20,525 bp) (Lindsey et al., 1989; Toba et al., 2011), Rac (22,890 bp) (Kaiser and Murray, 1979), Qin (20,456 bp) (Kaiser, 1980), e14 (15,204 bp) (Hiom et al., 1991; Mehta et al., 2004), and CPZ-55 (6,802 bp) prophages are well characterized. Several physiological functions such as stress tolerance, biofilm formation, antibiotic resistance, and other advantages to the host genome are attributed to the cryptic prophages in *E. coli* (Wang et al., 2010). These grounded prophages were suggested as potential drug targets (Wang and Wood, 2016). Analysis of locus-specific prophage sequences in completely sequenced *Escherichia* and *Shigella* strains (*E. coli* MG1655 strain as the reference) illustrates the significance of prophages in bacterial genome evolution and ecology at the level of a species. Examining the alignment of the specific locus in strains with and without prophage led to deduce the probable ancestral type locus and decipher the variations caused due to prophage integration. The tRNA genes are hotspots of phage integration based on sequence homology between the phage genome and tRNA gene (Campbell, 2003) and has been shown in case of integration of DLP12 prophage near the 3′ end of *argU* gene encoding tRNA-Arg. The probable recombinant *attR* site is similar to 47 bp of the *argU* gene, which was located on the other end of the prophage. The site-specific integration of e14 within the *icd* gene coding for isocitrate dehydrogenase protein (416aa) separated the 163 bp region from 3′ end of *icd* transcript. The integrated prophage provides the 3’ terminal codon of the *icd* ORF and thus forming homologous *attL* and *attR* sites [Supplementary Table S1 (Datasheet S1)]. Hence, the excision of e14 prophage results in restoration of functional *icd* gene yet rendering conservative replacement of aspartate by glutamate encoding codon (Hill et al., 1989). The site-specific integration of Rac prophage at the 5′ end of *ttcA* gene via the *attB* and *attP* sites resulted in the recombinant *attL* and *attR* sites. Variations in the amino acid sequence of the N-terminus of TtcA protein [a 311aa tRNA-cytidine(32) 2-sulfurtransferase protein involved in post-transcriptional thiolation of tRNA] was observed within the strains harboring Rac prophage. Such variations owing to sequence dissimilarity between the recombinant *attR* and *attL* sites reduce the fitness level in carbenicillin positive niche. The excision of Rac enhances biofilm formation, cell lysis, and motility (Hong et al., 2010; Liu et al., 2015). Integration of CPZ-55 prophage has occurred right after the stop codon of the *eutA* gene coding for ethanolamine utilization protein, causing target site duplication of 8 bp sequence (5′-TCAGGAAG-3′). The intergenic region of 13 bp (5′-TAAGTCGTTCCCT-3′) was conserved. No variation observed in prophage flanking genes, *eutB* (453 codons) and *eutA* (467 codons). However, CPZ-55 integration has separated the genes *eutA* and *eutB* and thereby disrupting the functionality of *eut* operon encoding proteins such as EutS, EutQ, EutN, EutD, EutM, EutE, EutH, and others, that are involved in ethanolamine reduction followed by utilization when available as the only nitrogen source. As the initial steps in the utilization of ethanolamine requires the expression of *eutA*, *eutB*, and *eutC*, any alterations as caused by prophage integration would result in the inability of *eut*^-^ strains to multiply upon utilizing ethanolamine (Soupene et al., 2003) (Figure 4) [Supplementary Table S1 (Datasheet S1)].

**FIGURE 5:**
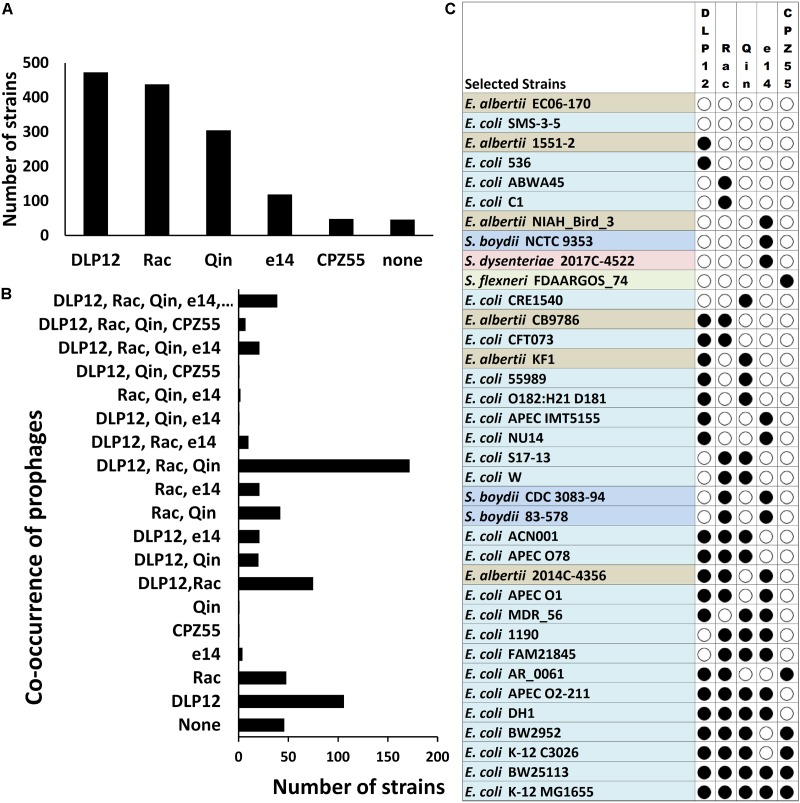
Locus-specific distribution of the five prophages (with reference to *E. coli* MG1655 strain) in 638 completely sequenced *E. coli* strains. The cryptic prophages (DLP12, e14, Rac, CPZ-55, and Qin) of MG1655 strain were analyzed for their distribution within 638 completely sequenced *E. coli* strains taking the bacterial and prophage borders sequences as a query in BLASTn. Only cryptic prophages of MG1655 strain with location specificity were analyzed. Other strains may have various other prophages at multiple locations. **(A)** The prevalence of each cryptic prophage within various *Escherichia* and *Shigella* strains. The distribution was determined by curating the BLAST hits obtained in completely sequenced strains showing >85% sequence identity. **(B)** The prevalence of various permutations of prophage co-occurrence within various *Escherichia* and *Shigella* strains. **(C)** The diversity in the locus-specific prophage distribution across *Escherichia* and *Shigella* species. Only representative strains are indicated for illustration purposes.

We analyzed the distribution of the selected cryptic prophages within different *Escherichia* and *Shigella* strains varies. Locus-specific sequence analysis of the above mentioned five prophages (loci as per *E. coli* MG1655) within the completely sequenced *Escherichia* and *Shigella* strains (a total of 638 strains) shows that DLP12 is the most prevalent (74%) prophage followed by Rac prophage in 69% of total strains (Figure 5A). Qin prophage is present in 48%, and e14 prophage is encoded by 19%, while only 8% of total strains encoded CPZ-55 prophage. The prevalence in the *E. coli* genomes indicates that the integration of DLP12 and Rac have happened earlier relative to the other prophages analyzed here. Forty-six strains did not harbor any of the five prophages scored for. The analysis of co-occurrence of prophages within *Escherichia* and *Shigella* strains would shed some light on the acquisition of the prophages during the evolution of these bacteria. Hence, we analyzed the co-occurrence of the five prophages (DLP12, e14, Rac, CPZ-55 and Qin) with all possible permutations in 638 completely sequenced and annotated *Escherichia* and *Shigella* genomes (available as on March 2018) (Figure 5). Although there are 32 different permutations of the five prophages, only 19 combinations were observed in our analysis [Supplementary Table S3 (Datasheet S2)]. If prophages were acquired sequentially, it would be expected that the various observed permutations would have an incremental pattern of prophages within various genomes. 172 *E. coli* genomes had DLP12, Rac, and Qin cryptic prophages while 106 genomes had DLP12 prophage and none of the other four prophages. Thirty-nine *E. coli* genomes including most of the K12 strains had the DLP12, Rac, Qin, e14, and CPZ-55 permutation (Figure 5B). In essence, we did not notice any particular pattern that might imply serial acquisition of cryptic prophages. For example, DLP12 is present with various combinations of other prophages that were analyzed indicating random co-occurrence (Figure 5C). The observed permutations are mostly random which would mean that these cryptic prophages also propagated horizontally among the *E. coli* strains. Horizontal propagation is common among bacterial strains; however, the mechanism of HGT is most likely through conjugation considering the large size of the cryptic prophages.

From our analysis of the 36 genes of Qin prophage from *E. coli* MG1655 strain, genes like *hokD*, *relBE*, cold-shock genes (e.g., *cspI*, *cspB*, etc.), and regulators (e.g., *dicC*, *dicB*, etc.) were predicted to have been acquired via HGT [Supplementary Table S2 (Datasheet S1)]. In our sequence analyses, we found multiple mutations in *intQ’* of most Qin prophage hits, which is indicative that the inactivation of the integrase gene could be a reason for the grounding of the Qin prophage. Although several Qin prophage genes are traditionally associated with general phage genes, numerous genes associated with Qin prophages are obtained by HGT. Several genes from plasmids and bacterial genomes (predominantly from *E. coli*, *E. albertii*, and *Citrobacter*) have integrated into the Qin prophage. The ancestral *hokD* ORF which is about 70 codons, however, is only 51 codons due to the integration of the *relBE* Toxin-Antitoxin system which is acquired from plasmids (Ramisetty and Santhosh, 2016). This observation emphasizes cautions to be exercised during characterization of genes that are highly linked to or within prophages because of high probability for genetic alterations. Comparisons of sequences of the same gene across several strains should be made to determine any truncations or modifications induced due to multiple genetic changes.

**FIGURE 6:**
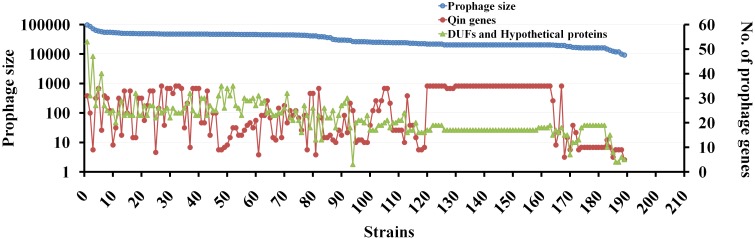
The genetic diversity of Qin prophage in various *E. coli* strains. Correlation between the prophage size and the number of genes carried within the prophage is studied using BLAST tool and curated in excel. For each strain, the length of site- specific Qin prophage and the number of prophage encoding proteins were obtained using BLASTn tool taking gene sequence of each gene as query and hits are curated using excel. The dependence of prophage size and the flux of genes are represented in a scatter plot as shown taking prophage size on primary *Y*-axis, the number of genes on the prophage as secondary *Y*-axis and 189 strains on the *X*-axis. For Qin prophage analyses, the prophage coordinates were used to estimate the prophage size. Having *E. coli* MG1655 as the reference strain, the number of genes carried on Qin prophage among 189 strains was noted. The number of DUFs (Domain of Unknown Function) and hypothetical proteins in Qin prophage of each strain was fetched using a JavaScript to search the GenBank files of each prophage for “DUF” and “hypothetical proteins.” The graph was plotted to present the diversity of prophage in terms of its size, the number of genes carried, and novel genes in each of the strains.

We then performed a comparative analysis of Qin prophage diversity within various *Escherichia* strains by estimating the length of prophage in base pairs (distance between the *att* sites), the number of Qin genes (with reference to MG1655 strain), and the number of *de novo* ORFs using a semantic script (hypothetical genes and DUFs). The size of the Qin prophage in 189 strains was highly divergent ranging from 11,280 bp in *E. coli* O6:H6 strain M9682-C1 to 97,450 bp in *E. coli* O26:H11 str. 11368 DNA (Figure 6). Surprisingly, we noticed a slight variation in Qin prophage size (of difference ∼1,300 bp) even within K-12 strains, which is indicative that prophages are highly prone to mutations. The number of copies of phage genes encoding major and minor capsid proteins, tail measure protein, host specificity protein, transposase, and other phage-related genes increased with the size of the Qin prophage. This observation indicates that cryptic prophages are hotspots for superintegration of phage/plasmid genomes (Lichens-Park et al., 1990). Conceivably, a positive correlation between the size of the prophage and the number of hypothetical proteins can be found. Some of the hypothetical genes could have accumulated as more plasmid/phage genes integrated into the Qin prophage. Nevertheless, there is a high probability for the generation of ORFs *de novo* due to multiple recombination events (Delaye et al., 2008; Tautz and Domazet-Lošo, 2011). Although the expressivity and functionality of most of the genes that originated through *de novo* genesis are unclear, they are genes in the making which maybe already functional or are yet to evolve for a beneficial function in the future generations.

To predict the possible role of the cryptic prophage in contributing to the ecological advantages to the host bacterium, we manually scouted for genes that are unrelated to the phage/plasmid in the Qin prophages of various *E. coli* strains [Supplementary Table S3 (Datasheet S2)]. The Qin prophage from strains examined carried diverse genes involved in significant functions such as transportation (e.g., Calcium transporter (*chaC*) in *E. coli* strain CRE1540), metabolism (e.g., Acyl-CoA thioesterase in *E. coli* O111:H-str. 11128 DNA), recombination (e.g., RusA endodeoxyribonuclease in *E. coli* strain ETEC-2265), virulence (e.g., Shiga toxin Stx2 subunits A and B in *E. coli* O178:H19 strain 2012C-4431), transcriptional regulation (e.g., LysR transcriptional regulator in *E. coli* strain CRE1540), and resistance (e.g., Tellurite/Colicin resistance protein in *E. coli* SE11 DNA) [Supplementary Table S3 (Datasheet S2)]. Using a semantic script, a total of 4,034 hypothetical proteins/DUFs were found in Qin prophages of 189 *Escherichia* and *Shigella* genomes indicating an average of 21 hypothetical genes per Qin prophage. Even after discounting 50% of the hypothetical genes for annotation and functionality issues, the high density and diversity of the hypothetical genes per prophage is indicative that grounded prophages are sites of *de novo* genesis which could expedite genome diversification and evolution (Berglund et al., 2010). In essence, grounded prophages of *E. coli* are hotspots of multiple recombination events, genetic flux, and *de novo* genesis of genes which expedite adaptations to various habitats.

## Conclusion

**FIGURE 7:**
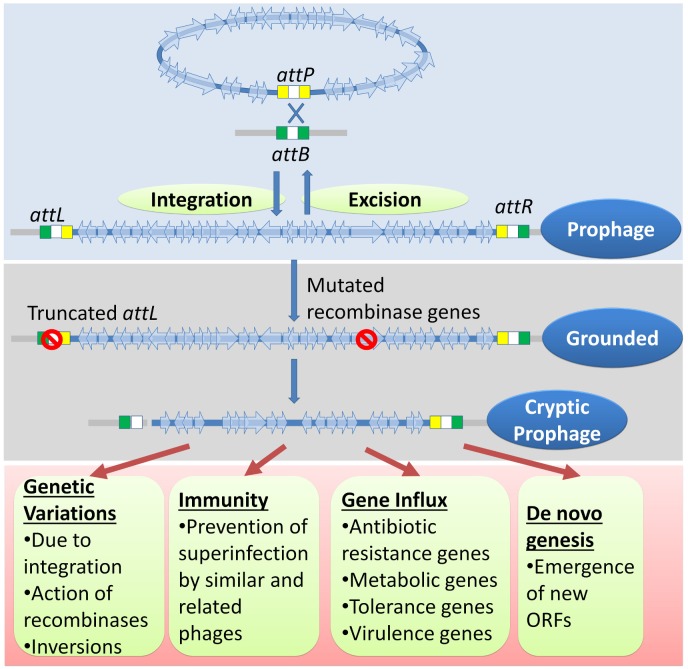
A genetic history and advantages of cryptic prophages. The genome of temperate phages integrates with the bacterial chromosome through site-specific recombination between sites similar to those of *attP* and *attB*. Occasionally, mutations in the *attL*, *attR*, recombinase genes, and genes encoding other essential accessory factors would result in a ‘grounded’ prophage. Eventually but gradually, more mutations, usually deletions, accumulate to ease the metabolic and genetic burden imposed by the cryptic prophage. The cryptic prophage offers several advantages to the host bacterium. The genetic variations caused in the host genome due to integrations, inversions involving prophage sequences, and the action of their recombinases may have a selective advantage. The prophage confers immunity from secondary phage infection and acts as hotspots for gene influx through horizontal gene transfer of important genes such as those involved in metabolism, antibiotic resistance, virulence, and stress tolerance. More importantly, the cryptic prophage is a site for the emergence of new ORFs that may have novel and useful functions.

The physiology of higher organisms is vastly influenced by their respective microbiomes. Microbiomes, in turn, are influenced by their phages (Scanlan, 2017; De Sordi et al., 2018). Bacteriophages are the major biological drivers of bacterial ecology and evolution through strategies such as symbiosis, dependency, and dormancy (Roossinck, 2011; Nasir et al., 2017). The prevalence of multiple grounded prophages within most bacterial genomes implies that the sequence of events such as lysogenization, grounding of prophages, domestication of phage genes, and the genetic events within the grounded prophages have an evolutionary impact on the selection of bacterial genomes. (1) Lysogeny, as opposed to the lytic cycle, represents an ‘evolutionarily stable strategy’ between bacteria and phages growing in an econiche to allow the existence of both the phage and the host. (2) The selection of lysogens is favored by the elimination of intraspecies competition by the phage and the immunity conferred to lysogens by the prophages. (3) The lysogens with grounded prophages are selected over lysogens (with wild-type prophages) because the latter have an imminent threat of prophage activation followed by lysis. The grounded prophages may provide immunity from specific phage attack, ecologically significant traits, and the evolution of new genes (Figure 7). Many genes that provide stress tolerance, antibiotic resistance, virulence genes, and metabolic genes acquired horizontally are located within prophages. (4) Grounded prophages are zones within genomes that allow multiple recombinations and mutations with minimal deleterious effects on the host physiology. The increased probability of genetic modifications (insertions, deletions, and inversions) within the prophages increases the probability of formation of new ORFs. These *de novo* genes maybe refined gradually for a function with a selective advantage such as antibiotic resistance and virulence.

Since the evolution of the first temperate phages, prophages must have enhanced the rate of evolution in different life forms including higher organisms. Grounded prophages are the most significant drivers of ecology, genetic diversification, and microbial genome evolution. The grounded prophages, and the analogous elements in other cellular life-forms, are the ‘holy grails’ of *de novo* genesis of genetic information, which must be of immense interest to molecular evolutionists.

## Author Contributions

BCMR has conceived the idea, designed the work, performed the analyses, interpreted the results, and wrote the manuscript. PAS has performed the work, interpreted the results, and wrote the manuscript.

## Conflict of Interest Statement

The authors declare that the research was conducted in the absence of any commercial or financial relationships that could be construed as a potential conflict of interest.
