# Supplementary material for: Bacterial ‘Grounded’ Prophages: Hotspots for Genetic Renovation and Innovation
Source: Front Genet. 2019 Feb 12;10:65. doi: 10.3389/fgene.2019.00065 (PMC6379469; doi:10.3389/fgene.2019.00065)
Supplement: Supplementary file 1 [file Data_Sheet_1.docx]

**Table S1: Properties of *E. coli* MG1655 prophages and the variations caused.**

| **Prophage** | **Size in bp** | **Variations due to prophage integration** | ***att* site** | **Excision** |
| --- | --- | --- | --- | --- |
| DLP12 | 20525 | No variations found  3' CCA is conserved | *attR*: 5'CTTCTAAGTCGTGGGCCGCAGGTTCGAATCCTGCAGGGCGCGCCATT3'  *attL*: 5'CTTCTAAGTCGTGGGCCGCAGGTTCGAATCCTGCAGGGCGCGCCATT3'  Identical repeat sequence | Irreversible ([X. Wang et al., 2010](#_ENREF_118)) |
| Rac | 22890 | Variation in the regulatory region of *ttcA*gene | *attR*: 5'TTGTTCAGGTTGTATTGTTCTTTCTTACTAATTTCTTGATTTTGCGACATT3'  *attL*: 5'TTGTTCAGGTTGTATTGTTCTTTCTTACTAATTTGTTGATTTTCTTGCATT3'  Non identical | Reversible ([Liu et al., 2015](#_ENREF_67)) |
| CPZ-55 | 6802 | Disruption of *eut* operon by separating *eutA* and *eutB* | TSD: 5'TCAGGAAG3'  Identical repeat sequence | Irreversible ([X. Wang et al., 2010](#_ENREF_118)) |
| e14 | 15204 | Conservative replacement of aspartate by glutamate amino acid in Icd protein | *attR*: 5'CTGCGCCACATGGGTTGGACCGAAGCGGCTGACTTAATTGTTAAAGGTATGGAAGGCGCAATCAACGCGAAAACCGTAACCTATGACTTCGAGCGTCTGATGGATGGCGCTAAACTGCTGAAATGTTCAGAGTTTGGTGACGCGATCATCGAAAACATGTAAT3'  *attL*: 5'CTGCGCCATATGGGTTGGACTGAAGCGGCTGACCTGATTGTTAAAGGTATGGAAGGCGCAATCAATGCCAAGACCGTAACTTATGACTTCGAACGTCTGATGGAAGGCGCTAAGCTGCTGAAATGTTCAGAGTTTGGTGAAGCGATCATCGAAAACATGTAAT3'  Non identical | Reversible ([Brody, Greener, & Hill, 1985](#_ENREF_14); [Brody & Hill, 1988](#_ENREF_15); [Kazuhiro, Toshifumi, & Tetsuo, 1985](#_ENREF_58); [Preeti Mehta1, 2014](#_ENREF_87)) |
| Qin | 20456 | Truncation of YdfJ protein (427 aa); lacking 28 aa (MTIEKHERSTKDLVKAAVSGWLGTALEF) on N terminal of YdfJ protein | TSD: 5'GAAATCCATAA3'  Identical repeat sequence | Irreversible ([X. Wang et al., 2010](#_ENREF_118)) |

**Method:**

**Determination of diversity within Qin prophage of *E. coli* strains:** Using the prophage coordinates the size of the prophage was calculated. Having *E. coli* MG1655 as the reference strain, the number of genes carried on Qin prophage among 189 strains was noted. The number of DUFs (Domain of Unknown Function) and hypothetical proteins in Qin prophage of each strain was fetched using Java script to search the Genbank files of each prophage for “DUF” and “hypothetical proteins”. The graph was plotted to present the diversity of prophage in terms of its size, the number of genes carried, and novel genes in each of the strains.

**Table S2. Genetic ontology of Qin prophage genes in *E. coli* MG1655.**

| **Best hits** | **Source** | **Qin proteins** | **Protein encoded** | **Gene conservation in percentage** |
| --- | --- | --- | --- | --- |
|  | Phage/Plasmid | YdfK | Cold shock protein | 87.38 |
|  | Plasmid | PinQ | DNA invertase | 83.98 |
|  | Phage related | TfaQ | Tail protein | 79.61 |
|  | Phage related | StfQ | Side-tail fiber protein | 30.10 |
|  | Phage related | NohQ | DNA packaging gene-pseudogene | 86.41 |
|  | Plasmid/Phage | YnfO | Uncharacterised | 56.31 |
|  | Phage related | YdfO | DUF1398 family protein | 46.60 |
|  | Undetermined | GnsB | sec**G** null mutant **s**uppressor **B** | 69.90 |
| *E. coli* | Bacterial | YnfN | Cold shock-induced protein | 69.42 |
|  | Bacterial/Plasmid | CspI | Cold-shock protein | 70.39 |
| *E. coli, Citrobacter amalonaticus* | Bacterial | RzpQ | Rz-like equivalent/host cell lysis? | 69.42 |
|  | Phage related | RrrQ | Lysozyme | 60.68 |
|  | Bacterial? Phage? | YdfR | DUF1327 family protein | 60.68 |
|  | Phage related | EssQ | Holin | 56.31 |
|  | Bacterial/plasmid | CspB | Cold-shock | 56.31 |
|  | Bacterial/Plasmid | CspF | Cold-shock | 51.46 |
|  | Bacterial/phage | QuuQ | Antiterminator | 64.56 |
|  | Bacterial/plasmid | YdfU | DUF968 family protein | 67.48 |
|  | Undetermined | Gene19 | Hypothetical | 71.84 |
|  | Undetermined | Rem | Putative regulator of exponential growth motility | 74.27 |
|  | Plasmid | HokD | Small toxic polypeptide | 96.12 |
|  | Plasmid | RelE | Endoribonuclease | 46.60 |
|  | Plasmid | RelB | Bifunctional antitoxin/transcriptional  repressor RelB | 46.60 |
| *S. enterica, Edwardsiella tarda, Enterobacter ludwigii* | Bacterial | FlxA | Uncharacterised | 48.06 |
|  | Bacterial/plasmid | Gene25 | Transposase | 46.12 |
| *E. alberti* | Bacterial | YdfX | Pseudogene | 70.39 |
| *E. alberti* | Bacterial | DicC | Transcriptional regulator | 39.32 |
| *E. alberti* | Bacterial | DicA | Transcriptional regulator | 37.38 |
| *E. coli, E. alberti* | Bacterial | YdfA | DUF1391 family protein | 71.84 |
| *E. coli, E. alberti* | Bacterial | YdfC | Uncharacterised | 64.08 |
| *E. coli, E. alberti* | Bacterial | Gene31 | Hypothetical | 53.88 |
| *E. coli* | Bacterial | DicB | Cell division inhibitor protein | 90.29 |
| *E. alberti* | Bacterial | YdfD | DUF1482 family protein, lysis protein | 88.35 |
| *E. alberti* | Bacterial | Gene34 | Exonuclease VIII | 85.92 |
|  | Bacterial/Plasmid | Transposase | Transposase |  |
| *E. alberti* | Bacterial | IntQ | Integrase-pseudogene | 95.63 |
